# Feelings first? Sex differences in affective and cognitive processes in emotion recognition

**DOI:** 10.1177/17470218211064583

**Published:** 2021-12-27

**Authors:** Judith Bek, Bronagh Donahoe, Nuala Brady

**Affiliations:** Perception Lab, School of Psychology, University College Dublin, Dublin, Ireland

**Keywords:** Emotion recognition, dynamic expressions, empathy, sex differences

## Abstract

The recognition of emotional expressions is important for social understanding and interaction, but findings on the relationship between emotion recognition, empathy, and theory of mind, as well as sex differences in these relationships, have been inconsistent. This may reflect the relative involvement of affective and cognitive processes at different stages of emotion recognition and in different experimental paradigms. In this study, images of faces were morphed from neutral to full expression of five basic emotions (anger, disgust, fear, happiness, and sadness), which participants were asked to identify as quickly and accurately as possible. Accuracy and response times from healthy males (*n* = 46) and females (*n* = 43) were analysed in relation to self-reported empathy (Empathy Quotient; EQ) and mentalising/theory of mind (Reading the Mind in the Eyes Test). Females were faster and more accurate than males in recognising dynamic emotions. Linear mixed-effects modelling showed that response times were inversely related to the emotional empathy subscale of the EQ, but this was accounted for by a female advantage on both measures. Accuracy was unrelated to EQ scores but was predicted independently by sex and Eyes Test scores. These findings suggest that rapid processing of dynamic emotional expressions is strongly influenced by sex, which may reflect the greater involvement of affective processes at earlier stages of emotion recognition.

## Introduction

The study of emotions, and their expression and recognition, is integral to the broader study of social cognition. In particular, emotion recognition—the ability to accurately read information about the emotional state of a conspecific from the face, voice, and body—confers survival value in both humans and non-human social species (Ferretti & Papaleo, 2019). By some accounts, the ability to empathise with others originates in more primitive and unconscious emotional processes, including motor contagion that reflects shared neural representations of perception and action (de Waal, 2012; Panksepp, 2011). Yet, despite the importance of emotion recognition and empathy, the relationship between them, and the influence of sex differences within this, are still poorly understood.

### Emotion recognition as an embodied process

The accurate and fast recognition of emotions from others’ facial expressions is important for effective social interaction and communication (Blair, 2003). It has been proposed that emotions expressed through the face are recognised through a process of embodied simulation (Gallese, 2005), whereby others’ expressions activate corresponding sensorimotor representations in the observer’s brain, and this is supported by recent neuroimaging evidence (Volynets et al., 2020). The embodied response may involve mimicry, which is the subthreshold activation of facial muscles involved in producing the target expression, as demonstrated in electromyography (EMG) studies (Sato et al., 2008). Consequently, it has also been proposed that sensorimotor processing deficits may contribute to impaired emotion recognition ability in conditions such as Parkinson’s disease (Ricciardi et al., 2017) and autism (Eigsti, 2013).

The majority of previous research into facial emotion recognition has used static images, which lack ecological validity given the dynamic nature of social interactions. When moving stimuli are used instead, emotion recognition tends to be faster and more accurate (Krumhuber et al., 2013). Electrophysiological and neuroimaging studies have found increased facial mimicry for dynamic expressions (Sato et al., 2008), as well as greater recruitment of sensorimotor and emotion-related brain regions (Arsalidou et al., 2011; Kessler et al., 2011), suggesting that simulation may be enhanced for dynamic stimuli. In addition, the use of motion cues in dynamic emotion recognition has been found to be altered in people with Parkinson’s disease, possibly reflecting reduced motor simulation (Bek et al., 2020).

### Affective and cognitive processes in emotion recognition

The ability to recognise and respond appropriately to emotional and mental states is considered to be a key aspect of empathy, which is more broadly defined as a set of skills necessary for relating to others (Baron-Cohen & Wheelwright, 2004; Christov-Moore et al., 2014; Decety & Jackson, 2006). Empathy has been suggested to involve both affective and cognitive components (Baron-Cohen & Wheelwright, 2004; Decety & Moriguchi, 2007). The affective component (sometimes termed “emotional empathy”) refers to the pre-cognitive processing of emotional states, and has been described as the vicarious sharing of emotion (Smith, 2006), implying a role of embodied simulation in this aspect of empathy. While Blair (2005) proposed a three-component structure of empathy, which additionally includes “motor empathy,” this third component can be seen to overlap with the concept of embodiment within affective empathy. Cognitive empathy, by contrast, is proposed to be a more explicit process of interpreting others’ behaviour in terms of their beliefs and intentions (Zaki & Ochsner, 2012). The term “cognitive empathy” has also been used synonymously or interchangeably with “theory of mind” and “mentalising” (Baron-Cohen et al., 2001; Lawrence et al., 2004). It has been argued that the affective and cognitive components of empathy work interactively rather than being separable processes or skills (Baron-Cohen & Wheelwright, 2004; Decety & Moriguchi, 2007). However, theory of mind and empathy have elsewhere been described as distinct social-cognitive processes (e.g., Fortier et al., 2018), and emotional and cognitive aspects of empathy may recruit different neural systems, the former being associated with regions involved in motor simulation and mirroring (Shamay-Tsoory, 2011; Zaki & Ochsner, 2012).

Although emotion recognition has been extensively studied in different populations, with deficits found across a number of developmental, psychiatric, and neurodegenerative conditions alongside other social-cognitive impairments (Christidi et al., 2018; Elamin et al., 2012; Harms et al., 2010; Kohler et al., 2003), its relationship with empathy is not well understood. There is some evidence associating emotion recognition with self-report empathy measures such as the Empathy Quotient (EQ; Baron-Cohen & Wheelwright, 2004), which is a widely used and validated instrument designed to measure cognitive and affective facets of empathy in both research and clinical settings. Scores on the EQ as well as the “empathic concern” subscale of the Interpersonal Reactivity Index (IRI; Davis, 1983) have been found to be related to recognition of basic emotions from static expressions (Besel & Yuille, 2010; Sucksmith et al., 2013), and the IRI has been associated with recognition of dynamic expressions (Lewis et al., 2016). Other studies have shown a positive association of EQ scores with accuracy in imitating emotional facial expressions (Williams et al., 2013), and an inverse relationship with neural activity during a dynamic emotion perception task (Chakrabarti et al., 2006).

The Reading the Mind in the Eyes test (Eyes Test; Baron-Cohen et al., 2001) is a widely used task that assesses the ability to infer complex emotions and other mental states from photographs of the eye region of human faces. Although the Eyes Test was designed to assess cognitive empathy, mentalising, or theory of mind, it has also been described as an emotion recognition test (Alaerts et al., 2011; Oakley et al., 2016; Vellante et al., 2013), and has been found to correlate with measures of emotional processing, including “emotional intelligence” (Megías-Robles et al., 2020) and emotion recognition (Hargreaves et al., 2016; Henry et al., 2009; Olderbak et al., 2015; Petroni et al., 2011). A positive correlation between the EQ and Eyes Test has been found (Lawrence et al., 2004; Voracek & Dressler, 2006), which may reflect the involvement of cognitive aspects of empathy in this task. However, this has not consistently been replicated (Baron-Cohen et al., 2015; Vellante et al., 2013).

The heterogeneity among tasks and stimuli used to assess emotion recognition presents a particular challenge to understanding its relationship with aspects of empathy. Previous studies of facial emotion recognition have differed in terms of stimulus and task characteristics (see meta-analysis by Thompson & Voyer, 2014), which may tap into different mechanisms, potentially accounting for some of the inconsistencies in previous findings. For example, it has been proposed that affective processes may have a greater involvement in the more automatic recognition of emotional expressions at shorter exposures, whereas at longer exposures cognitive strategies may be invoked (Besel & Yuille, 2010).

### Sex differences in empathy and emotion recognition

Given the role of emotional processes in social understanding and interaction, it is important to consider how sex differences may influence emotion recognition and empathy. It is likely that there are multiple contributors to such differences, including genetic, biochemical, and environmental factors (Christov-Moore et al., 2014; Kret & De Gelder, 2012). Within an evolutionary framework of empathy, for example, maternal instincts including sensitivity to emotional signals from offspring have particular significance (e.g., de Waal, 2012; Panksepp, 2011), prompting the study of sex differences in emotion recognition (e.g., Hampson et al., 2006) and in affective neuroscience more generally (Kret & De Gelder, 2012).

Typically, females score higher than males on the EQ and other self-report empathy scales (Baez et al., 2017; Baron-Cohen & Wheelwright, 2004; Greenberg et al., 2018; Kidron et al., 2018; Lawrence et al., 2004; Vellante et al., 2013). Sex differences in relation to the different components of empathy have also been suggested; for example, neuroimaging during empathy tasks has revealed that females showed stronger activations in areas associated with emotional processing (including the amygdala), while males showed greater activation of cognitive processing areas (Derntl et al., 2010).

Females are also generally faster and more accurate in recognising emotional expressions (see Kret & De Gelder, 2012; Thompson & Voyer, 2014) and notably, females show a greater advantage than males in recognising dynamic compared with static emotions (e.g., Biele & Grabowska, 2006). Differences between males and females in neural activations (Li et al., 2020; Stevens & Hamann, 2012) and eye gaze patterns (Hall et al., 2010; Vassallo et al., 2009) during emotion recognition tasks have suggested that there may be sex differences in underlying mechanisms. In addition, in EMG studies, females have shown increased facial mimicry of emotional expressions, and appear to utilise feedback from facial muscles more than males during emotion recognition (Sonnby-Borgström et al., 2003; Stel & Van Knippenberg, 2008). Together with the clearer female advantage in recognising dynamic stimuli, this suggests a greater role of embodied simulation in females, which might correspond to processing emotions at a more automatic and affective level (Christov-Moore et al., 2014). A recent meta-analysis of 28 studies (Holland et al., 2021) found a significant overall relationship between various self-report empathy measures and facial mimicry of emotions in females but not males across both static and dynamic paradigms. Although there was no significant relationship between mimicry and emotion recognition, this additional analysis only included a subset of nine studies and response times (RTs) were not examined, potentially obscuring a role of mimicry in the early processing of emotions.

In contrast to tasks using dynamic emotion stimuli, the Eyes Test, which as noted above involves identifying complex emotions and mental states from static expressions, is assumed to reflect more cognitive aspects of empathy, and has not consistently shown superior performance by females (Alaerts et al., 2011; Baron-Cohen et al., 2015; Dodell-Feder et al., 2020; Olderbak et al., 2015; Vellante et al., 2013). Previous studies have also failed to find a clear role of sex differences in the relationship between empathy and static emotion recognition. For example, sex did not influence the prediction of Eyes Test scores by dimensions of emotional intelligence (Megías-Robles et al., 2020), and was not found to moderate the relationship between self-reported empathy and recognition of static emotions (Besel & Yuille, 2010).

### The present study

The aim of this study was to clarify the relationship between recognition of dynamic emotional expressions and empathy, and to examine how sex differences may influence this relationship. Females were expected to show higher scores on self-reported empathy (EQ), particularly in affective/emotional empathy, and to be faster and more accurate in recognising dynamic emotional expressions. Dynamic emotion recognition was expected to be related to levels of self-reported empathy, but based on previous findings it was not clear whether this relationship would be influenced by sex differences. In addition, associations between emotion recognition, empathy, sex, and recognition of complex emotions/mental states from static expressions (using the Eyes Test) were explored.

## Methods

### Participants

A convenience sample of 89 students (43 females, 46 males) from University College Dublin (UCD) participated in the study, with a mean age of 22.7 years (*SD* = 5.2), which did not differ significantly between females and males, *t*(87) = 0.57; *p* = .57; Cohen’s *d* = 0.12. Participants reported no significant history of neurological or psychiatric conditions. The study was approved by the UCD Human Research Ethics Committee and participants provided informed consent.

### Procedure and materials

Tasks were completed in the following fixed order: (1) dynamic emotion recognition, (2) complex emotion/mental state recognition (Eyes Test), (3) self-reported empathy (EQ). The dynamic emotion recognition task was administered using Presentation (Neurobehavioral Systems, Inc.) and the Eyes Test and EQ were administered in Qualtrics (Provo, UT). Stimuli were presented on a Dell XPS-8300 PC with a screen size of 19 inches and display resolution of 2048 × 1152.

#### Dynamic emotion recognition

The emotion recognition task was based on Lynch et al. (2006). Participants observed a series of faces that morphed from a neutral expression to a full emotional expression and judged which emotion was being expressed. The stimuli were created using images from the Stirling/ESRC 3D Face Database.^1^ Six expressions (anger, disgust, fear, happiness, sadness, and neutral) posed by six different actors (three males, three females) were selected. Surprise was not included in the set of emotions because pilot work indicated that it could not be reliably differentiated from expressions of fear, and surprise is suggested to be a more complex emotion that may require additional cognitive processing (Baron-Cohen et al., 2008). The face morphing package Psychomorph^2^ was used to transform each face from neutral to full emotional expression (Tiddeman et al., 2005) using a guide by Sutherland (2015). Each face was morphed from 100% neutral to 100% emotion in 21 steps with a 5% increase in the emotional expression at each step. For each face and emotion, the 21 morphs (rendered in eight-bit colour) were used to create.avi format videos using iMovie. The video frames subtended ~11.5 by ~10.9 degrees of visual angle at a comfortable viewing distance of ~60 cm.

**Figure 1.**
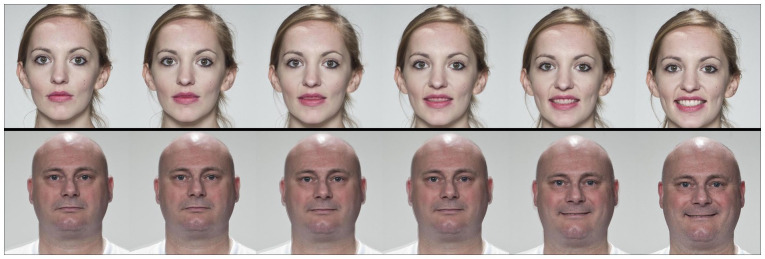
Examples of face images (comparable to those used in the dynamic emotion recognition task) showing the morphing from neutral (far left) through to full expression (far right) in increments of 20% for the emotion of happiness. Morph examples are for illustrative purposes only, created using WebMorph (DeBruine, 2018) with images available under CC BY license from DeBruine & Jones (2017).

Examples of the stimuli are shown in Figure 1. In each trial, participants watched a short video (8,500 ms) of the face morphing from neutral to full expression and indicated which of the five emotions was displayed, using a Cedrus Response Box labelled with the corresponding emotion words. Participants were advised to respond as quickly as possible once they were reasonably confident as to the emotion displayed; i.e., both speed and accuracy were emphasised. Following the final frame, participants were given a further opportunity to select an emotion based on a single presentation of a static image showing the full expression of the emotion (100%); however, responses were very similar to the initial emotion judgements and are not reported further. The task consisted of 30 test trials, which were randomised across the six actors and six expressions. This was preceded by a demo trial showing a face morphing from neutral to full expression, and two practice trials, which used different stimuli to those in the test trials.

#### Empathy Quotient

The EQ (Baron-Cohen & Wheelwright, 2004) is a self-report questionnaire requiring participants to rate their agreement with each of 40 statements (e.g., “Seeing people cry doesn’t really upset me”; “I am good at predicting how someone will feel”) on a 4-point scale. Responses of “*agree*” or “*strongly agree*” score one or two points, respectively, while “*disagree*” and “*strongly disagree*” are both scored as zero, resulting in a total score out of 80. Approximately half of the items are reverse-scored.

#### Eyes Test

The Reading the Mind in the Eyes Test—Revised (Eyes Test; Baron-Cohen et al., 2001) is a test of complex emotion and mental state recognition, in which participants are presented with 36 photographs of the eye region of male and female actors’ faces and select which word (out of four options) best describes what the person is thinking or feeling. A glossary is provided in case participants are unfamiliar with any of the mental state terms.

#### Data analysis

Based on factor analysis by Muncer and Ling (2006; also see Groen et al., 2015), the EQ was divided into three subscales, each comprising five items, which are assumed to reflect “cognitive empathy,” “emotional empathy,” and “social skills.” Statistical analysis was conducted in R (R Core Team, 2021).

Initial analysis of RTs and accuracy (percent correct) for each emotion in the dynamic recognition task in males and females was conducted using multiple regression. Sex differences on the EQ and Eyes Test were analysed using independent *t*-tests, and Pearson’s correlation coefficients between the EQ and Eyes Test were calculated. Linear mixed-effects modelling was then conducted using the R package lme4 (Bates et al., 2015), to further explore sex differences and relationships with the EQ and Eyes Test.

## Results

**Figure 2.**
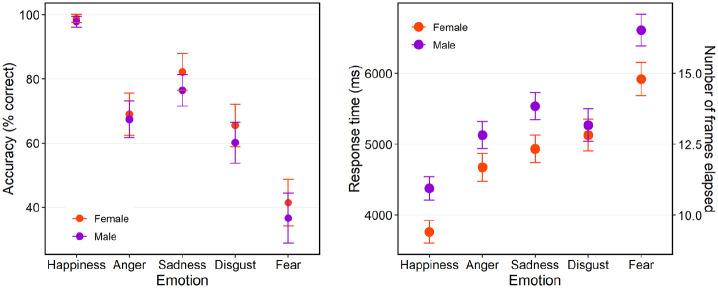
Accuracy (percent correct; left) and response times/number of frames elapsed to correctly identify each of the five emotions (right) by females and males. Error bars show 95% confidence intervals.

Figure 2 shows accuracy (percent correct) in recognising the five different emotions and RTs with the corresponding number of frames elapsed to correctly identify the target emotion. RTs were naturally limited at the upper end as the task timed out if participants did not respond by the end of the morph sequence. RTs were shortest for happiness and longest for fear, and there was no evidence of a speed/accuracy trade-off, with accuracy being highest for happiness and lowest for fear. Females appeared faster and more accurate than males, with a more pronounced sex difference for RTs. Multiple regression analyses confirmed these observations (Table 1): relative to the baseline emotion of happiness, RTs increased and accuracy decreased for all other emotions, and males were slower and less accurate than females.

**Table 1.** Multiple regression analysis of effects of each of the five basic emotions and sex on RT and accuracy in the dynamic emotion recognition task, showing coefficient estimates, *t*-values and significance levels (significant factors shown in bold).

Predictor	RT (ms)			*R*^2^/*R*^2^-adjusted	Accuracy (% correct)			*R*^2^/*R*^2^-adjusted
	Estimate	*t*	*p*		Estimate	*t*	*p*	
(Intercept)	3,819.10	56.19	**<.001**		100.23	44.86	**<.001**	
Emotion: fear	2,192.42	19.58	**<.001**		−59.36	−20.70	**<.001**	
Emotion: sadness	1,164.16	13.04	**<.001**		−19.10	−6.66	**<.001**	
Emotion: disgust	1,129.56	11.82	**<.001**		−35.58	−12.41	**<.001**	
Emotion: anger	831.13	8.92	**<.001**		−30.15	−10.51	**<.001**	
Sex: male	503.20	7.93	<**.001**		−3.71	−2.05	**.041**	
				.212/.210				.516/.510

### Empathy Quotient

**Figure 3.**
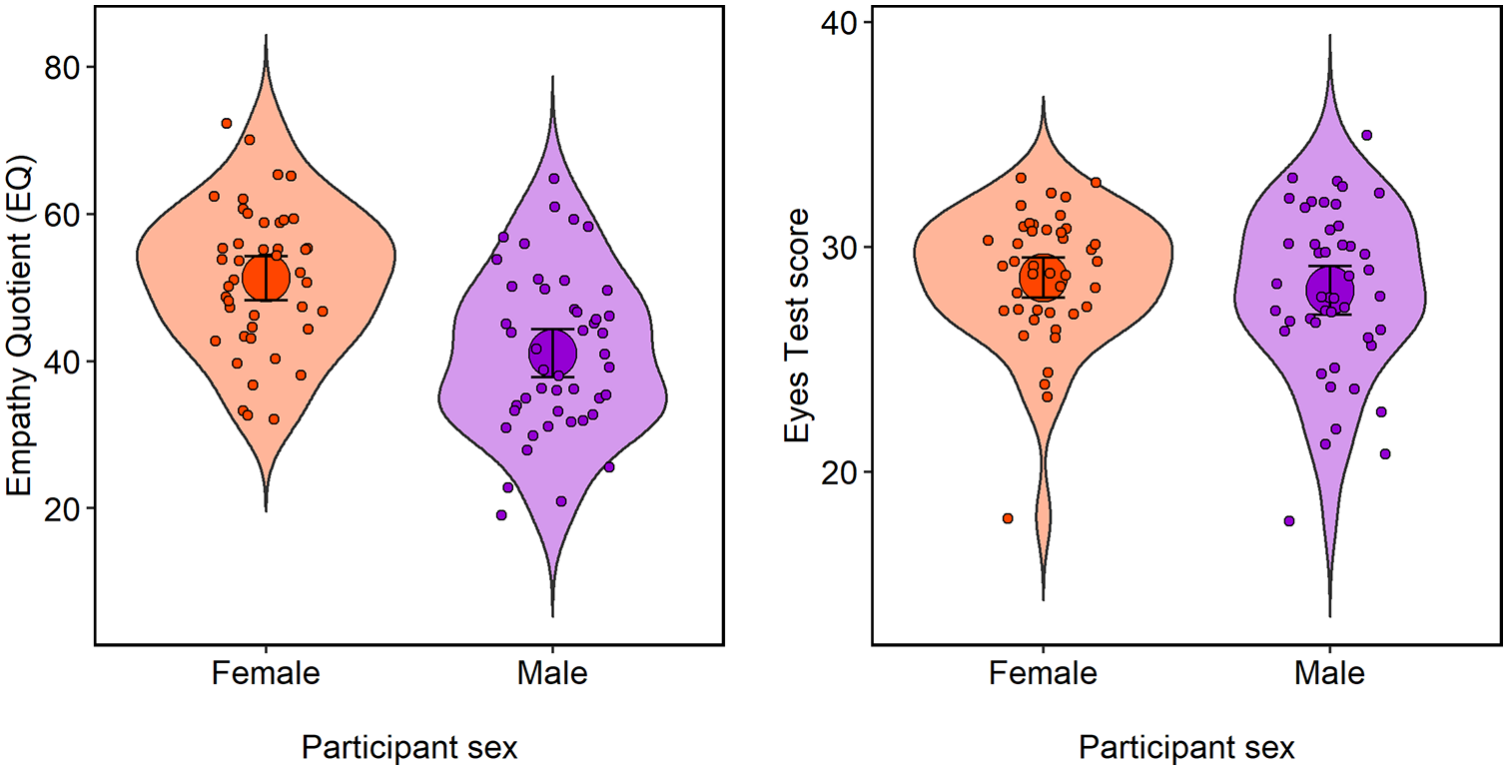
Violin plots showing the mean EQ score (left) and mean Eyes Test score (right) with 95% confidence intervals. Dots represent individual data points.

The distribution of scores on the EQ is shown in the left panel of Figure 3. Females scored significantly higher than males on the EQ total score, females *M* = 51.28, males *M* = 41.04, *t*(87) = −4.65, *p* < .001; Cohen’s *d* = 0.99. Females also scored significantly higher than males on the cognitive empathy subscale, *t*(87) = −2.77, *p* = .006; Cohen’s *d* = 0.59, and the emotional empathy subscale, *t*(87) = −6.51, *p* < .001; Cohen’s *d* = 1.38, but the difference on the social skills subscale was much less marked, *t*(87) = −1.76, *p* = .08; Cohen’s *d* = 0.37.

### Eyes Test

**Figure 4.**
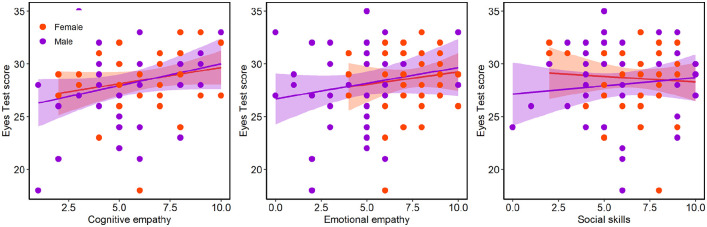
EQ scores were positively correlated with Eyes Test scores for the total scale and the cognitive empathy subscale, but significant correlations were not found for the emotional empathy or social skills subscales.

As illustrated in the right panel of Figure 3, there was no significant sex difference for the Eyes Test, females *M* = 28.63, males *M* = 28.07; *t*(87) = −0.80, *p* = .43; Cohen’s *d* = 0.17. A significant positive correlation between the Eyes Test and the EQ was found across all participants, *r*(87) = 0.31; *p* = .003, as well as for females, *r* (41) = .31; *p* = .045. Correlations between the Eyes test and each subscale of the EQ were also analysed. As illustrated in Figure 4, there was a significant relationship with cognitive empathy, *r*(87) = .26; *p* = .013, but the correlation with emotional empathy did not reach significance, *r*(87) = .19; *p* = .08, and there was no evidence of a correlation with social skills, *r*(87) = .052; *p* = .63. Correlations were not significant when females and males were analysed separately.

### Predictors of dynamic emotion recognition

**Figure 5.**
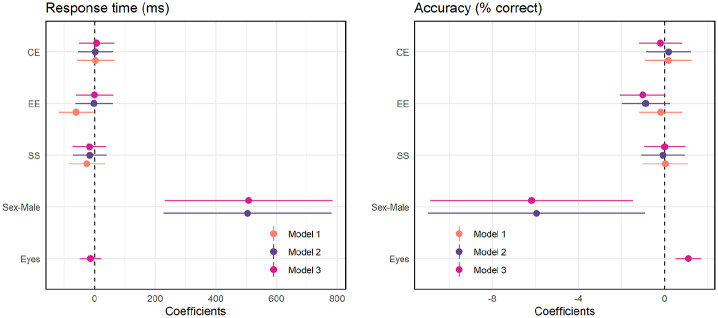
Dot-and-whisker plots showing regression coefficients with 95% confidence intervals for each of the three linear mixed models in predicting RT (left) and accuracy (right). Model 1 includes the EQ subscales as fixed factors; sex is added into Model 2 and Eyes Test scores are added into Model 3. Model 2 appears to provide the best fit for RT and Model 3 fits best for accuracy. While the addition of sex increased the power of both models, the effect appears to be greater for RT than accuracy.

In modelling RT, the intercepts for participants, stimuli, and emotions were included as random effects. Predictor variables were added successively into models as fixed factors (see Table 2 and Figure 5). Model 1 included the three EQ subscales, showing a significant effect of the emotional empathy subscale, with higher scores predicting faster RTs. Model 2 included sex as an additional factor, showing a significant effect whereby males were slower than females, but the effect of emotional empathy became non-significant. In Model 3, scores on the Eyes Test were added in, which showed no significant effect. Comparing the models with likelihood ratio tests showed that the inclusion of sex added explanatory power, Model 1 vs. Model 2; χ^2^(1) = 12.0; *p* = .0005, but Eyes Test scores did not, Model 2 vs. Model 3; χ^2^(1) = 0.50; *p* = .48. The effect of sex in Model 2 likely masks that of emotional empathy because the latter is largely attributable to sex differences in empathy. It is noted that the coefficient for sex is much larger because this represents the estimated difference in the dependent variable (RT) between groups, while those for the EQ subscales and Eyes Test represent the estimated difference in RT for a one-point change in EQ or Eyes Test score.

**Table 2.** Results of linear mixed modelling to predict RT and accuracy in the dynamic emotion recognition task, showing fixed factors included in each iteration of the model, with coefficient estimates, *t*-values and significance levels (significant fixed factors in each model shown in bold).

RT (ms)					Accuracy (% correct)			
Predictor	Estimate	*t*	*p*	Marginal/conditional *R*^2^	Estimate	*t*	*p*	Marginal/conditional *R*^2^
Model 1								
(Intercept)	5,647.31	13.51	**<** **.001**		69.32	7.24	**<.001**	
EQ-CE	4.06	0.13	.896		0.17	0.31	.754	
EQ-EE	−60.23	−2.10	**.036**		−0.18	−0.36	.719	
EQ-SS	−25.04	−0.82	.410		0.03	0.06	.949	
				.011 / .382				.000 / .557
Model 2								
(Intercept)	4,978.99	11.04	**<.001**		77.19	7.63	**<.001**	
EQ-CE	2.94	0.10	.920		0.19	0.35	.725	
EQ-EE	−1.46	−0.05	.963		−0.87	−1.52	.129	
EQ-SS	−14.95	−0.52	.601		−0.08	−0.16	.875	
Sex: male	504.85	3.58	**<.001**		−5.95	−2.31	**.021**	
				.028 / .382				.008 / .557
Model 3								
(Intercept)	5,311.85	8.15	**<.001**		48.67	3.81	**<.001**	
EQ-CE	7.33	0.25	.805		−0.19	−0.37	.709	
EQ-EE	0.28	0.01	.993		−1.02	−1.89	.058	
EQ-SS	−16.09	−0.56	.573		0.01	0.02	.987	
Sex: male	508.20	3.61	**<.001**		−6.18	−2.57	**.010**	
Eyes Test	−12.82	−0.71	.480		1.10	3.56	<**.001**	
				.028 / .382				.025 / .557

EQ: empathy quotient; CE: cognitive empathy; EE: emotional empathy; SS: social skills; Eyes Test: total score on Reading the Mind in the Eyes Test.

In modelling the accuracy data, the intercepts for participants and emotions were included as random effects. Fixed effects were entered into subsequent models in the same order as for RT. As shown in Table 2 (and Figure 5), there was no significant effect of the EQ subscales in Model 1. Adding sex into Model 2 showed that males were significantly less accurate than females, and adding Eyes Test scores into Model 3 resulted in a significant effect associating higher scores with higher accuracy in dynamic emotion recognition, while retaining a significant effect of sex. Likelihood ratio tests showed that each subsequent model added explanatory power, Model 1 versus Model 2, χ^2^(1) = 5.19; *p* = .023; Model 2 versus Model 3, χ^2^(1) = 11.68; *p* = .00057, such that sex and Eyes Test both independently contributed to predicting accuracy.

## Discussion

This study examined the relationship between recognition of dynamic emotional expressions and empathy, as well as the role of sex differences within this. In line with previous findings (e.g., Beaudry et al., 2014; Ekman & Friesen, 1976; Lynch et al., 2006), happiness was the most easily identified expression while fear was the most difficult, and females were faster and more accurate than males in recognising emotions (e.g., Kret & De Gelder, 2012; Thompson & Voyer, 2014), although the evidence was stronger for RTs than accuracy. Females also showed higher levels of self-reported empathy on the EQ, as previously reported (e.g., Baron-Cohen & Wheelwright, 2004; Greenberg et al., 2018).

Linear mixed modelling was used to explore the contributions of different aspects of empathy and sex differences in predicting performance on the emotion recognition task. Although faster RTs were associated with higher scores on the emotional empathy subscale of the EQ, this effect was found to be accounted for by sex differences in the two measures. Higher scores on the EQ have previously been associated with earlier recognition of morphed emotional expressions (Kosonogov et al., 2015), but the influence of sex within this relationship was not reported. It is also possible that sex differences contributed to other previous findings associating self-reported empathy with dynamic emotion recognition (e.g., Lewis et al., 2016).

Given that responses tend to be faster and more accurate to dynamic than static stimuli (Krumhuber et al., 2013), and that increased mimicry has been found for moving expressions (Rymarczyk et al., 2016; Sato et al., 2008), it follows that dynamic emotion recognition tasks may recruit automatic, affective processes to a greater extent than static tasks. Moreover, the female advantage in emotion recognition appears to be greater for dynamic than static stimuli, as indicated by evidence from intensity ratings (Biele & Grabowska, 2006), empathic responses (Kuypers, 2017), and mimicry (Rymarczyk et al., 2016). The shorter RTs exhibited by females in this study as well as in previous studies may, therefore, relate to a greater involvement of affective processing, which is also reflected in their higher emotional empathy scores. This suggestion is also consistent with previous research indicating greater recruitment of emotion-related brain regions during empathy tasks in females (Derntl et al., 2010).

There is also some evidence associating emotion recognition at briefer presentation durations with more affective aspects of empathy, as measured by the “empathic concern” subscale of the IRI (Besel & Yuille, 2010). As noted above for dynamic emotions, mimicry has also been associated with recognition of briefly presented (500 ms) stimuli (Borgomaneri et al., 2020), and a recent meta-analysis found a stronger relationship between empathy and mimicry of emotions at shorter stimulus durations (Holland et al., 2021). If there is an increased reliance on affective processing at shorter stimulus exposure durations, it might be expected that sex differences would also be amplified. Indeed, it has been suggested that inconsistent findings on sex differences in neural activations during processing of emotional stimuli may be, in part, accounted for by the involvement of different processes at different durations (Kret & De Gelder, 2012). However, studies using static emotion recognition paradigms have not found differential effects of sex at different latencies (e.g., Hall & Matsumoto, 2004). Also using static stimuli, Besel and Yuille (2010) found that sex did not influence either the relationship between emotion recognition and affective empathy (“empathic concern”) at a shorter presentation duration (50 ms) or the relationship with the EQ at a longer duration (2,000 ms).

To further understand the roles of sex differences and empathy in different aspects of emotion recognition, this study also explored how dynamic emotion recognition and empathy might relate to performance on the Eyes Test, which has been described both as a test of theory of mind or mentalising (Baron-Cohen et al., 2001) and a test of complex emotion recognition (e.g., Oakley et al., 2016). Performance on the Eyes Test was associated with accuracy but not RTs in dynamic emotion recognition, which was independent of sex differences. The embodied simulation of observed expressions might be greatly reduced in the Eyes Test, as the stimuli are static and only show a limited portion of the face. This would be expected to increase reliance on top–down inferential processing, thus attenuating the female advantage typically found in emotion recognition. The absence of a significant sex difference in the Eyes Test in this study is consistent with previous findings from a large sample of participants (Olderbak et al., 2015).

Accuracy in the Eyes Test correlated positively with the cognitive empathy subscale of the EQ, which also fits with the suggestion that the processing of emotional expressions at later stages corresponds more closely to higher level or cognitive aspects of empathy (e.g., Besel & Yuille, 2010). The relationship of cognitive empathy with accuracy in the Eyes Test but not in the dynamic emotion recognition task could reflect the more complex nature of the emotions and mental states in the Eyes Test. However, while some previous studies have found a relationship between the Eyes Test and EQ (Lawrence et al., 2004; Voracek & Dressler, 2006), others have not (Baron-Cohen et al., 2015; Vellante et al., 2013), and Lawrence et al. (2004) found neither EQ nor sex to significantly predict Eyes Test scores. Interpretation of performance on the Eyes Test has also been noted to be complicated by its reliance on verbal ability (Lawrence et al., 2004; Olderbak et al., 2015), and the influence of education, race, and ethnicity (Dodell-Feder et al., 2020).

Finally, the absence of an independent relationship between empathy scores on the EQ subscales and dynamic emotion recognition requires further investigation. Although Muncer and Ling (2006) found their subscales to have adequate reliability and validity, further psychometric analysis has suggested that the EQ measures a single construct of empathy (Allison et al., 2011), while others have argued that the overall scale reflects cognitive more than affective aspects of empathy (Besel & Yuille, 2010; Dziobek et al., 2008).

More broadly, the inconsistent terminology and definitions used to describe emotion recognition, theory of mind and empathy, and the measures used to test these constructs, make it difficult to identify clear relationships between different facets of social-cognitive processing (as noted by Mitchell & Phillips, 2015). Future research should more systematically investigate the relationships of dynamic and static emotion recognition with different empathy measures and sex differences, using carefully designed paradigms permitting the measurement of accuracy, RTs, and mimicry.

This study provides further evidence on sex differences in dynamic emotional processing, with a larger sample size than previous studies (e.g., Biele & Grabowska, 2006), while also indicating how sex differences may influence the relationship between emotional processing and empathy. Nonetheless, some limitations should be acknowledged when interpreting the findings and designing future studies. The dynamic stimuli in this study were created by morphing still frames of posed expressions, which may have resulted in less naturalistic portrayal than if spontaneous expressions were used, and consequently may have increased the difficulty of the task. In addition, this study did not include a direct comparison of dynamic and static versions of the same expressions, which may have enabled stronger conclusions to be drawn. It could also be argued that requiring participants to make an emotion judgement while the expression was morphing required the recruitment of additional cognitive processes.

In conclusion, the present findings suggest that the female advantage in speed of identifying emotions from dynamic expressions may reflect the involvement of more automatic affective processes at earlier stages of emotion recognition. The recognition of emotions at longer durations, or from more complex static expressions, may increasingly involve top–down inferential processing, and appears to be less susceptible to sex differences. The influence of sex on emotion recognition and empathy may reflect an evolutionary adaptation (e.g., Hampson et al., 2006), such that the faster processing of basic emotional states by females alongside affective empathy may have arisen from primary caretaking roles. The present findings also suggest a mechanism by which interpersonal understanding and behaviour might differ between males and females in dynamic social scenarios.

## Notes

1.
http://pics.psych.stir.ac.uk/ERSC/

2.
http://users.aber.ac.uk/bpt/jpsychomorph/

## References

[bibr1-17470218211064583] Alaerts K. Nackaerts E. Meyns P. Swinnen S. P. Wenderoth N. (2011). Action and emotion recognition from point light displays: An investigation of gender differences. PLOS ONE, 6, Article e20989. 10.1371/journal.pone.0020989 PMC3111458 21695266

[bibr2-17470218211064583] Allison C. Baron-Cohen S. Wheelwright S. J. Stone M. H. Muncer S. J. (2011). Psychometric analysis of the Empathy Quotient (EQ). Personality and Individual Differences, 51(7), 829–835. 10.1016/j.paid.2011.07.005.

[bibr3-17470218211064583] Arsalidou M. Morris D. Taylor M. J. (2011). Converging evidence for the advantage of dynamic facial expressions. Brain Topography, 24, 149–163. 10.1007/s10548-011-0171-4 21350872

[bibr4-17470218211064583] Baez S. Flichtentrei D. Prats M. Mastandueno R. García A. M. Cetkovich M. Ibáñez A. (2017). Men, women . . .who cares? A population-based study on sex differences and gender roles in empathy and moral cognition. PLOS ONE, 12, Article e0179336. 10.1371/journal.pone.0179336 PMC5478130 28632770

[bibr5-17470218211064583] Baron-Cohen S. Bowen D. C. Holt R. J. Allison C. Auyeung B. Lombardo M. V. Smith P. Lai M. C. (2015). The “reading the mind in the eyes” test: Complete absence of typical sex difference in 400 men and women with autism. PLOS ONE, 10, Article e0136521. 10.1371/journal.pone.0136521 PMC4552377 26313946

[bibr6-17470218211064583] Baron-Cohen S. Spitz A. Cross P. (2008). Do children with autism recognise surprise? A research note. Cognition and Emotion, 7(6), 507–516. 10.1080/02699939308409202

[bibr7-17470218211064583] Baron-Cohen S. Wheelwright S. (2004). The empathy quotient: An investigation of adults with Asperger syndrome or high functioning autism, and normal sex differences. Journal of Autism and Developmental Disorders, 34(2), 163–175. 10.1023/b:jadd.0000022607.19833.00 15162935

[bibr8-17470218211064583] Baron-Cohen S. Wheelwright S. Hill J. Raste Y. Plumb I. (2001). The “Reading the Mind in the Eyes” test revised version: A study with normal adults, and adults with Asperger syndrome or high-functioning autism. Journal of Child Psychology and Psychiatry and Allied Disciplines, 42(2), 241–251. 10.1017/s0021963001006643 11280420

[bibr9-17470218211064583] Bates D. Maechler M. Bolker B. Walker S. (2015). Fitting Linear Mixed-Effects Models Using lme4. Journal of Statistical Software, 67(1), 1–48. 10.18637/jss.v067.i01.

[bibr10-17470218211064583] Beaudry O. Roy-Charland A. Perron M. Cormier I. Tapp R. (2014). Featural processing in recognition of emotional facial expressions. Cognition and Emotion, 28, 416–432. 10.1080/02699931.2013.833500 24047413

[bibr11-17470218211064583] Bek J. Poliakoff E. Lander K. (2020). Measuring emotion recognition by people with Parkinson’s disease using eye-tracking with dynamic facial expressions. Journal of Neuroscience Methods, 331, Article 108524. 10.1016/j.jneumeth.2019.108524 31747554

[bibr12-17470218211064583] Besel L. D. S. Yuille J. C. (2010). Individual differences in empathy: The role of facial expression recognition. Personality and Individual Differences, 49, 107–112. 10.1016/j.paid.2010.03.013

[bibr13-17470218211064583] Biele C. Grabowska A. (2006). Sex differences in perception of emotion intensity in dynamic and static facial expressions. Experimental Brain Research, 171, 1–6. 10.1007/s00221-005-0254-0 16628369

[bibr14-17470218211064583] Blair R. J. R. (2003). Facial expressions, their communicatory functions and neuro-cognitive substrates. Philosophical Transactions of the Royal Society B: Biological Sciences, 358, 561–572. 10.1098/rstb.2002.1220 PMC1693136 12689381

[bibr15-17470218211064583] Blair R. J. R. (2005). Responding to the emotions of others: Dissociating forms of empathy through the study of typical and psychiatric populations. Consciousness and Cogni-tion, 14(4), 698–718. 10.1016/J.CONCOG.2005.06.004 16157488

[bibr16-17470218211064583] Borgomaneri S. Bolloni C. Sessa P. Avenanti A. (2020). Blocking facial mimicry affects recognition of facial and body expressions. PLOS ONE, 15, Article e0229364. 10.1371/journal.pone.0229364 PMC7032686 32078668

[bibr17-17470218211064583] Chakrabarti B. Bullmore E. Baron-Cohen S. (2006). Empathizing with basic emotions: Common and discrete neural substrates. Social Neuroscience, 1, 364–384. 10.1080/17470910601041317 18633800

[bibr18-17470218211064583] Christidi F. Migliaccio R. Santamaría-García H. Santangelo G. Trojsi F. (2018). Social cognition dysfunctions in neurodegenerative diseases: Neuroanatomical correlates and clinical implications. Behavioural Neurology, 2018, Article 1849794. 10.1155/2018/1849794 PMC5944290 29854017

[bibr19-17470218211064583] Christov-Moore L. Simpson E. A. Grigaityte K. Iacoboni M. Ferrari P. F. Sciences B. Behavior H. Angeles L. Shriver K. (2016). Empathy: Gender effects in brain and behaviour. Neuroscience and Biobehavioral Reviews, 46, 604–627. 10.1016/j.neubiorev.2014.09.001.Empathy PMC5110041 25236781

[bibr20-17470218211064583] Davis M. H. (1983). Measuring individual—differences in empathy—Evidence for a multidimensional approach. Journal of Personality and Social Psychology, 44(1), 113–126. 10.1037//0022-3514.44.1.113

[bibr21-17470218211064583] DeBruine L. (2018). WebMorph (Beta Release 2). Zenodo. 10.5281/zenodo.1073696.

[bibr22-17470218211064583] DeBruine L. Jones B. (2017). Jones. 2017. Face Research Lab London Set. figshare. 10.6084/m9.figshare.5047666.v3

[bibr23-17470218211064583] Decety J. Jackson P. L. (2006). A social-neuroscience perspective on empathy. Current Directions in Psychological Science, 15(2), 54–58. 10.1111/j.0963-7214.2006.00406.x

[bibr24-17470218211064583] Decety J. Moriguchi Y. (2007). The empathic brain and its dysfunction in psychiatric populations: Implications for intervention across different clinical conditions. BioPsychoSocial Medicine, 1, Article 22. 10.1186/1751-0759-1-22 PMC2206036 18021398

[bibr25-17470218211064583] Derntl B. Finkelmeyer A. Eickhoff S. Kellermann T. Falkenberg D. I. Schneider F. Habel U. (2010). Multidimensional assessment of empathic abilities: Neural correlates and gender differences. Psychoneuroendocrinology, 35(1), 67–82. 10.1016/J.PSYNEUEN.2009.10.006 19914001

[bibr26-17470218211064583] de Waal F. B. M. (2012). The antiquity of empathy. Science, 336(6083), 874–876. 10.1126/SCIENCE.1220999 22605767

[bibr27-17470218211064583] Dodell-Feder D. Ressler K. J. Germine L. T. (2020). Social cognition or social class and culture? On the interpretation of differences in social cognitive performance. Psychological Medicine, 50, 133–145. 10.1017/S003329171800404X 30616706

[bibr28-17470218211064583] Dziobek I. Rogers K. Fleck S. Bahnemann M. Heekeren H. R. Wolf O. T. Convit A. (2008). Dissociation of cognitive and emotional empathy in adults with Asperger syndrome using the Multifaceted Empathy Test (MET). Journal of Autism and Developmental Disorders, 38, 464–473. 10.1007/s10803-007-0486-x 17990089

[bibr29-17470218211064583] Eigsti I.-M. (2013). A review of embodiment in autism spectrum disorders. Frontiers in Psychology, 4, Article 224. 10.3389/fpsyg.2013.00224 PMC3639406 23641226

[bibr30-17470218211064583] Ekman P. Friesen W. V. (1976). Measuring facial movement. Environmental Psychology and Nonverbal Behavior, 1(1), 56–75. 10.1007/bf01115465

[bibr31-17470218211064583] Elamin M. Pender N. Hardiman O. Abrahams S. (2012). Social cognition in neurodegenerative disorders: A systematic review. Journal of Neurology, Neurosurgery and Psychiatry, 83, 1071–1079. 10.1136/jnnp-2012-302817 22869923

[bibr32-17470218211064583] Ferretti V. Papaleo F. (2019). Understanding others: Emotion recognition in humans and other animals. Genes, Brain and Behavior, 18(1), Article e12544. 10.1111/GBB.12544 30549185

[bibr33-17470218211064583] Fortier J. Besnard J. Allain P. (2018). Theory of mind, empathy and emotion perception in cortical and subcortical neurodegenerative diseases. Revue Neurologique, 174, 237–246. 10.1016/j.neurol.2017.07.013 29622366

[bibr34-17470218211064583] Gallese V. (2005). Embodied simulation: From neurons to phenomenal experience. Phenomenology and the Cognitive Sciences, 4, 23–48. 10.1007/s11097-005-4737-z

[bibr35-17470218211064583] Greenberg D. M. Warrier V. Allison C. Baron-Cohen S. (2018). Testing the empathizing–systemizing theory of sex differences and the extreme male brain theory of autism in half a million people. Proceedings of the National Academy of Sciences of the United States of America, 115, 12152–12157. 10.1073/pnas.1811032115 30420503 PMC6275492

[bibr36-17470218211064583] Groen Y. Fuermaier A. B. M. Den Heijer A. E. Tucha O. Althaus M. (2015). The empathy and systemizing quotient: The psychometric properties of the Dutch Version and a Review of the Cross-Cultural Stability. Journal of Autism and Developmental Disorders, 45, 2848–2864. 10.1007/s10803-015-2448-z 25911303 PMC4553147

[bibr37-17470218211064583] Hall J. K. Hutton S. B. Morgan M. J. (2010). Sex differences in scanning faces: Does attention to the eyes explain female superiority in facial expression recognition? Cognition and Emotion, 24, 629–637. 10.1080/02699930902906882

[bibr38-17470218211064583] Hall J. A. Matsumoto D. (2004). Gender Differences in Judgments of Multiple Emotions From Facial Expressions. Emotion, 4(2), 201–206. 10.1037/1528-3542.4.2.201 15222856

[bibr39-17470218211064583] Hampson E. van Anders S. M. Mullin L. I. (2006). A female advantage in the recognition of emotional facial expressions: Test of an evolutionary hypothesis. Evolution and Human Behavior, 27(6), 401–416. 10.1016/J.EVOLHUMBEHAV.2006.05.002

[bibr40-17470218211064583] Hargreaves A. Mothersill O. Anderson M. Lawless S. Corvin A. Donohoe G. (2016). Detecting facial emotion recognition deficits in schizophrenia using dynamic stimuli of varying intensities. Neuroscience Letters, 633, 47–54. 10.1016/j.neulet.2016.09.017 27637386

[bibr41-17470218211064583] Harms M. B. Martin A. Wallace G. L. (2010). Facial emotion recognition in autism spectrum disorders: A review of behavioral and neuroimaging studies. Neuropsychology Review, 20, 290–322. 10.1007/s11065-010-9138-6 20809200

[bibr42-17470218211064583] Henry J. D. Phillips L. H. Beatty W. W. McDonald S. Longley W. A. Joscelyne A. Rendell P. G. (2009). Evidence for deficits in facial affect recognition and theory of mind in multiple sclerosis. Journal of the International Neuropsychological Society, 15, 277–285. 10.1017/S1355617709090195 19203428

[bibr43-17470218211064583] Holland A. C. O’Connell G. Dziobek I. (2021). Facial mimicry, empathy, and emotion recognition: A meta-analysis of correlations. Cognition and Emotion, 35, 150–168. 10.1080/02699931.2020.1815655 32924798

[bibr44-17470218211064583] Kessler H. Doyen-Waldecker C. Hofer C. Hoffmann H. Traue H. C. Abler B. (2011). Neural correlates of the perception of dynamic versus static facial expressions of emotion. Psychosocial Medicine, 8, Article Doc03. 10.3205/psm000072 PMC3080662 21522486

[bibr45-17470218211064583] Kidron R. Kaganovskiy L. Baron-Cohen S. (2018). Empathizing-systemizing cognitive styles: Effects of sex and academic degree. PLOS ONE, 13, Article e0194515. 10.1371/journal.pone.0194515 PMC5868797 29579056

[bibr46-17470218211064583] Kohler C. G. Turner T. H. Bilker W. B. Brensinger C. M. Siegel S. J. Kanes S. J. Gur R. E. Gur R. C. (2003). Facial emotion recognition in schizophrenia: Intensity effects and error pattern. American Journal of Psychiatry, 160, 1768–1774. 10.1176/appi.ajp.160.10.1768 14514489

[bibr47-17470218211064583] Kosonogov V. Titova A. Vorobyeva E. (2015). Empathy, but not mimicry restriction, influences the recognition of change in emotional facial expressions. Quarterly Journal of Experimental Psychology, 68, 2106–2115. 10.1080/17470218.2015.1009476 25608032

[bibr48-17470218211064583] Kret M. E. De Gelder B. (2012). A review on sex differences in processing emotional signals. Neuropsychologia, 50, 1211–1221. 10.1016/j.neuropsychologia.2011.12.022 22245006

[bibr49-17470218211064583] Krumhuber E. G. Kappas A. Manstead A. S. R. (2013). Effects of dynamic aspects of facial expressions: A review. Emotion Review, 5, 41–46. 10.1177/1754073912451349

[bibr50-17470218211064583] Kuypers K. P. C. (2017). Emotional empathic responses to dynamic negative affective stimuli is gender-dependent. Frontiers in Psychology, 8, Article 1491. 10.3389/fpsyg.2017.01491 PMC5583588 28912745

[bibr51-17470218211064583] Lawrence E. J. Shaw P. Baker D. Baron-Cohen S. David A. S. (2004). Measuring empathy: Reliability and validity of the Empathy Quotient. Psychological Medicine, 34, 911–920. 10.1017/S0033291703001624 15500311

[bibr52-17470218211064583] Lewis G. J. Lefevre C. E. Young A. W. (2016). Functional architecture of visual emotion recognition ability: A latent variable approach. Journal of Experimental Psychology: General, 145, 589–602. 10.1037/xge0000160 26986040

[bibr53-17470218211064583] Li G. Zhang S. Le T. M. Tang X. Li C. S. R. (2020). Neural responses to negative facial emotions: Sex differences in the correlates of individual anger and fear traits. NeuroImage, 221, Article 117171. 10.1016/j.neuroimage.2020.117171 PMC7789231 32682098

[bibr54-17470218211064583] Lynch T. R. Rosenthal M. Z. Kosson D. S. Cheavens J. S. Lejuez C. W. Blair R. J. R. (2006). Heightened sensitivity to facial expressions of emotion in borderline personality disorder. Emotion, 6, 647–655. 10.1037/1528-3542.6.4.647 17144755

[bibr55-17470218211064583] Megías-Robles A. Gutiérrez-Cobo M. J. Cabello R. Gómez-Leal R. Baron-Cohen S. Fernández-Berrocal P. (2020). The “Reading the mind in the Eyes” test and emotional intelligence: Eyes Test and emotional intelligence. Royal Society Open Science, 7, Article 201305. 10.1098/rsos.201305rsos201305 PMC7540806 33047068

[bibr56-17470218211064583] Mitchell R. L. C. Phillips L. H. (2015). The overlapping relationship between emotion perception and theory of mind. Neuropsychologia, 70, 1–10. 10.1016/j.neuropsychologia.2015.02.018 25687032

[bibr57-17470218211064583] Muncer S. J. Ling J. (2006). Personality and individual differences—Psychometric analysis of the Empathy Quotient (EQ) scale. Personality and Individual Differences, 40, 1111–1119.

[bibr58-17470218211064583] Oakley B. F. M. Brewer R. Bird G. Catmur C. (2016). Theory of mind is not theory of emotion: A cautionary note on the reading the mind in the eyes test. Journal of Abnormal Psychology, 125, 818–823. 10.1037/abn0000182 27505409 PMC4976760

[bibr59-17470218211064583] Olderbak S. Wilhelm O. Olaru G. Geiger M. Brenneman M. W. Roberts R. D. (2015). A psychometric analysis of the reading the mind in the eyes test: Toward a brief form for research and applied settings. Frontiers in Psychology, 6, Article 1503. 10.3389/fpsyg.2015.01503 PMC4593947 26500578

[bibr60-17470218211064583] Panksepp J. (2011). The basic emotional circuits of mammalian brains: Do animals have affective lives? Neuroscience & Biobehavioral Reviews, 35(9), 1791–1804. 10.1016/J.NEUBIOREV.2011.08.003 21872619

[bibr61-17470218211064583] Petroni A. Canales-Johnson A. Urquina H. Guex R. Hurtado E. Blenkmann A. Von Ellenrieder N. Manes F. Sigman M. Ibañez A. (2011). The cortical processing of facial emotional expression is associated with social cognition skills and executive functioning: A preliminary study. Neuroscience Letters, 505, 41–46. 10.1016/j.neulet.2011.09.062 22001365

[bibr62-17470218211064583] R Core Team. (2021). R: A language and environment for statistical computing.

[bibr63-17470218211064583] Ricciardi L. Visco-Comandini F. Erro R. Morgante F. Bologna M. Fasano A. Ricciardi D. Edwards M. J. Kilner J. (2017). Facial emotion recognition and expression in Parkinson’s disease: An emotional mirror mechanism? PLOS ONE, 12, Article e0169110. 10.1371/journal.pone.0169110 PMC5221788 28068393

[bibr64-17470218211064583] Rymarczyk K. Zurawski Ł. Jankowiak-Siuda K. Szatkowska I. (2016). Do dynamic compared to static facial expressions of happiness and anger reveal enhanced facial mimicry? PLOS ONE, 11, Article e0158534. 10.1371/journal.pone.0158534 PMC4938565 27390867

[bibr65-17470218211064583] Sato W. Fujimura T. Suzuki N. (2008). Enhanced facial EMG activity in response to dynamic facial expressions. International Journal of Psychophysiology, 70, 70–74. 10.1016/j.ijpsycho.2008.06.001 18598725

[bibr66-17470218211064583] Shamay-Tsoory S. G. (2011). The neural bases for empathy. Neuroscientist, 17, 18–24. 10.1177/1073858410379268 21071616

[bibr67-17470218211064583] Smith A. (2006). Cognitive empathy and emotional empathy in human behavior and evolution. Psychological Record, 56, 3–21. 10.1007/BF03395534

[bibr68-17470218211064583] Sonnby-Borgström M. Jönsson P. Svensson O. (2003). Emotional empathy as related to mimicry reactions at different levels of information processing. Journal of Nonverbal Behavior, 27, 3–23. 10.1023/A:1023608506243

[bibr69-17470218211064583] Stel M. Van Knippenberg A. (2008). The role of facial mimicry in the recognition of affect. Psychological Science, 19, 984–985. 10.1111/j.1467-9280.2008.02188.x 19000207

[bibr70-17470218211064583] Stevens J. S. Hamann S. (2012). Sex differences in brain activation to emotional stimuli: A meta-analysis of neuroimaging studies. Neuropsychologia, 50, 1578–1593. 10.1016/j.neuropsychologia.2012.03.011 22450197

[bibr71-17470218211064583] Sucksmith E. Allison C. Baron-Cohen S. Chakrabarti B. Hoekstra R. A. (2013). Empathy and emotion recognition in people with autism, first-degree relatives, and controls. Neuropsychologia, 51, 98–105. 10.1016/j.neuropsychologia.2012.11.013 23174401 PMC6345368

[bibr72-17470218211064583] Sutherland C. A. M. (2015). A basic guide to psychomorph. University of York.

[bibr73-17470218211064583] Thompson A. E. Voyer D. (2014). Sex differences in the ability to recognise non-verbal displays of emotion: A meta-analysis. Cognition and Emotion, 28, 1164–1195. 10.1080/02699931.2013.875889 24400860

[bibr74-17470218211064583] Tiddeman B. P. Stirrat M. R. Perrett D. I. (2005). Towards realism in facial image transformation: Results of a wavelet MRF method. Computer Graphics Forum, 24(3), 449–456. 10.1111/j.1467-8659.2005.00870.x

[bibr75-17470218211064583] Vassallo S. Cooper S. L. Douglas J. M. (2009). Visual scanning in the recognition of facial affect: Is there an observer sex difference? Journal of Vision, 9(3), Article 11. 10.1167/9.3.11 19757950

[bibr76-17470218211064583] Vellante M. Baron-Cohen S. Melis M. Marrone M. Petretto D. R. Masala C. Preti A. (2013). The “reading the Mind in the Eyes” test: Systematic review of psychometric properties and a validation study in Italy. Cognitive Neuropsychiatry, 18, 326–354. 10.1080/13546805.2012.721728 23106125 PMC6345369

[bibr77-17470218211064583] Volynets S. Smirnov D. Saarimäki H. Nummenmaa L. (2020). Statistical pattern recognition reveals shared neural signatures for displaying and recognizing specific facial expressions. Social Cognitive and Affective Neuroscience, 15(8), 803–813. 10.1093/scan/nsaa110 33007782 PMC7543934

[bibr78-17470218211064583] Voracek M. Dressler S. G. (2006). Lack of correlation between digit ratio (2D:4D) and Baron-Cohen’s “Reading the Mind in the Eyes” test, empathy, systemising, and autism-spectrum quotients in a general population sample. Personality and Individual Differences, 41, 1481–1491. 10.1016/j.paid.2006.06.009

[bibr79-17470218211064583] Williams J. H. G. Nicolson A. T. A. Clephan K. J. de Grauw H. Perrett D. I. (2013). A novel method testing the ability to imitate composite emotional expressions reveals an association with empathy. PLOS ONE, 8, Article e61941. 10.1371/journal.pone.0061941 PMC3633957 23626756

[bibr80-17470218211064583] Zaki J. Ochsner K. (2012). The neuroscience of empathy: Progress, pitfalls and promise. Nature Neuroscience, 15, 675–680. 10.1038/nn.3085 22504346

